# Cell-type specific mechanisms of D-serine uptake and release in the brain

**DOI:** 10.3389/fnsyn.2014.00012

**Published:** 2014-05-30

**Authors:** Magalie Martineau, Vladimir Parpura, Jean-Pierre Mothet

**Affiliations:** ^1^Department of Cellular Biophysics, Institute for Medical Physics and Biophysics, University of MuensterMuenster, Germany; ^2^Department of Neurobiology, University of Alabama at BirminghamBirmingham, AL, USA; ^3^Department of Biotechnology, University of RijekaRijeka, Croatia; ^4^Aix Marseille University, CRN2M UMR7286 CNRSMarseille, France

**Keywords:** NMDA receptors, coagonists, exocytosis, transporters, calcium, synapse

## Abstract

Accumulating evidence during the last decade established that D-serine is a key signaling molecule utilized by neurons and astroglia in the mammalian central nervous system. D-serine is increasingly appreciated as the main physiological endogenous coagonist for synaptic NMDA receptors at central excitatory synapses; it is mandatory for long-term changes in synaptic strength, memory, learning, and social interactions. Alterations in the extracellular levels of D-serine leading to disrupted cell-cell signaling are a trademark of many chronic or acute neurological (i.e., Alzheimer disease, epilepsy, stroke) and psychiatric (i.e., schizophrenia) disorders, and are associated with addictive behavior (i.e., cocaine addiction). Indeed, fine tuning of the extracellular levels of D-serine, achieved by various molecular machineries and signaling pathways, is necessary for maintenance of accurate NMDA receptor functions. Here, we review the experimental data supporting the notion that astroglia and neurons use different pathways to regulate levels of extracellular D-serine.

## INTRODUCTION

**FIGURE 1:**
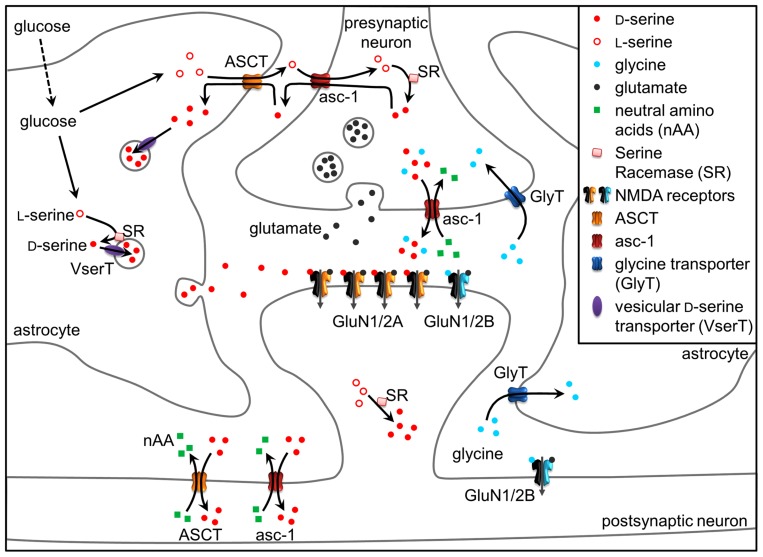
**D-Serine at the tripartite synapse.** L-Serine produced in astrocytes shuttles to neurons through alanine–serine–cysteine transporter 1 (asc-1) and fuels the synthesis of D-serine by SR. D-Serine in turn shuttles from neurons to astrocytes, where it accumulates in glial vesicles. Neuronal D-serine release may occur following depolarization through asc-1, while astroglial D-serine release may occur through exocytosis following the activation of receptors at the astrocyte plasma membrane. Once in the synaptic cleft, D-serine binds to synaptic NMDAR-containing GluN2A subunits. Conversely, extrasynaptic receptors containing GluN2B preferentially bind glycine, diffusion of which towards the cleft is prevented by GlyT1 transporters. Glycine released by neurons through asc-1 could, however, activate synaptic NMDARs to a lesser degree than D-serine. D-serine is finally removed from the synaptic cleft via ASCT and asc-1 transporters. See text for details.

*N*-methyl D-aspartate receptors (NMDARs) are glutamate-gated ionotropic receptors/channels that are central to many physiological processes including learning and memory, and are involved in neurotoxicity and psychiatric disorders (Traynelis et al., 2010; Paoletti et al., 2013). In addition to glutamate, NMDAR activation, i.e., channel opening, requires the binding of a coagonist, initially thought to be glycine (Johnson and Ascher, 1987; Kleckner and Dingledine, 1988; **Figure 1**). However, many studies during the last 15 years have shown that D-serine, an unusual amino acid synthesized in the brain by serine racemase (SR; Campanini et al., 2013) and degraded by the peroxysomal flavoprotein D-amino acid oxidase (DAAO; Sacchi et al., 2012), is the main coagonist of synaptic NMDARs in various brain areas (Martineau et al., 2006; Wolosker, 2011; Billard, 2012; Van Horn et al., 2013). Accordingly, D-serine is a physiological modulator of many NMDAR-dependent functions, including brain development (Kim et al., 2005), synaptic transmission and long-term synaptic plasticity (**Figure 1**; Mothet et al., 2000; Yang et al., 2003; Papouin et al., 2012; Li et al., 2013; Rosenberg et al., 2013), as well as learning, memory, and social interactions (Labrie et al., 2008; DeVito et al., 2011). Additionally, alterations in D-serine metabolism and extracellular levels appear to be central to several pathological states. For example, D-serine levels are greatly increased in the spinal cord of patients with amyotrophic lateral sclerosis and a mouse model of this disease, where it likely mediates motor neuron degeneration (Mitchell et al., 2010; Sasabe et al., 2012). Conversely, a decrease of D-serine levels would be associated with the hypofunction of NMDARs in schizophrenia (Sacchi et al., 2012; Balu et al., 2013), could be responsible for cognitive impairments observed during healthy aging (Billard, 2012), and for the behavioral hypersensitization to cocaine addiction (Curcio et al., 2013). Initially, the effects of D-serine in the brain had been attributed solely to its role as a gliotransmitter (Martineau et al., 2006), but this notion has been subsequently challenged by the discovery that D-serine is also produced and released by neurons (Wolosker et al., 2008). Albeit the various roles have been attributed to D-serine, the relative contribution of astrocytes and neurons in D-serine-mediated processes are still unclear. Due to two distinct cellular sources of this amino acid, the mechanisms underlying D-serine dynamics and the modulation of NMDAR activity are likely to be much more complex than previously assumed. As excellent reviews have been recently published on the biochemistry of SR (Campanini et al., 2013) and DAAO (Sacchi et al., 2012), the present review focuses primarily on the molecular entities used by neurons and astrocytes in regulation of D-serine functions.

## NEURONAL VERSUS ASTROGLIAL D-SERINE

The biosynthesis of D-serine was clarified in 1999 by the identification of SR that converts L-serine to D-serine (Wolosker et al., 1999). SR is a highly regulated enzyme that binds to several receptor-interacting proteins including GRIP1 (Kim et al., 2005), PICK1 (Hikida et al., 2008) and DISC1 (Ma et al., 2013), while it is inhibited by nitric oxide (Mustafa et al., 2007), palmitoylation-mediated membrane binding (Balan et al., 2009) and phosphatidylinositol(4,5)-biphosphate (Mustafa et al., 2009). SR knockout (SR-KO) mice exhibit less than 15% of normal forebrain D-serine levels and altered glutamatergic neurotransmission, supporting the notion that D-serine is a physiological NMDAR coagonist (Inoue et al., 2008; Basu et al., 2009; Horio et al., 2011). Initial studies localized D-serine and SR predominantly in astrocytes (Schell et al., 1997; Wolosker et al., 1999; Panatier et al., 2006) leading to the conclusion that D-serine would be preferentially a gliotransmitter. However, the subsequent use of new and more sensitive/specific antibodies against SR and SR-KO mice as negative controls indicate that SR prevails in excitatory and inhibitory neuronal populations throughout the brain (Kartvelishvily et al., 2006; Miya et al., 2008; Balu et al., 2014). Additionally, the generation of conditional cell-specific SR-KO mice reveals a lower forebrain D-serine level along with long-term potentiation (LTP) deficits when SR gene is deleted in neurons, while deletion in astrocytes leads to a minimal decrease in forebrain SR expression and no significant change in D-serine level and NMDAR activity (Benneyworth et al., 2012). Although neuronal SR-KO mice showed a strong reduction in SR expression in the forebrain they exhibit only a moderate decrease in D-serine level supporting the notion that peripheral (i.e., non-brain) sources may also provide for D-serine in the brain (Benneyworth et al., 2012).

Although neurons may appear to be the primary source of D-serine in the brain, this does not imply that astrocytes have only a minor role in D-serine mediated functions. In many brain regions, astrocytic D-serine is more abundant than neuronal D-serine (Kartvelishvily et al., 2006; Williams et al., 2006; Martineau et al., 2013 but see Balu et al., 2014). If neurons are the major source of *de novo* D-serine synthesis by SR, how would then D-serine be more abundant in astrocytes? The answer to this question may be provided by the elegant hypothesis of a serine shuttle between neurons and astrocytes (Wolosker, 2011). Namely, neuronal D-serine depends on the production of L-serine by astrocytes because of the virtually exclusive astrocytic localization of 3-phosphoglycerate dehydrogenase which catalyzes the production of L-serine from glucose (Yamasaki et al., 2001; Yang et al., 2010; Ehmsen et al., 2013). L-serine is then exported and shuttles to neurons to fuel the synthesis of D-serine by SR. D-serine is, in turn, released by neurons and accumulates back in astrocytes, where it is stored and released upon stimulation of astrocytes (Wolosker, 2011; **Figure 1**).

The notion that D-serine is a gliotransmitter is reinforced by many studies investigating its functions in synaptic transmission and plasticity. In an early study, LTP was evoked in cultured hippocampal neurons when they were co-cultured with astrocytes or supplemented with exogenous D-serine (Yang et al., 2003). In line with these data, the disruption of glial metabolism by the toxin fluoroacetate, in acute slice preparations of the medial prefrontal cortex or of the hippocampus, impaired NMDAR-mediated synaptic transmission and LTP induction by reducing the extracellular levels of D-serine (Henneberger et al., 2010; Fossat et al., 2012). The contribution of astroglial D-serine to activity-induced synaptic plasticity was further supported in a physiological model of astroglia-neuron structural plasticity in the hypothalamus (Panatier et al., 2006). Here, during lactation, the degree of astrocytic coverage of neuronal synapses decreased leading to a reduction in NMDAR-dependent synaptic transmission and the abolition of LTP. This phenotype was fully reversible when D-serine levels were exogenously restored. Conversely, lowering D-serine levels reduced synaptic transmission in virgin animals. These studies seem to establish the contribution of astroglia as the main source of D-serine supply for synaptic activity. This is further evidenced by the elegant study using a mouse model for the selective and inducible expression of a human mutant form of DISC1 in astrocytes (Ma et al., 2013). Mutant DISC1 does not bind SR and its expression down-regulates endogenous DISC1 leading to increased ubiquitination and degradation of SR in astrocytes and thus to reduced production of astroglial D-serine and to behavioral abnormalities consistent with hypofunction of NMDA neurotransmission.

Of course, neuron-derived D-serine takes part in NMDAR-mediated synaptic transmission and plasticity. Stimulation of D-serine release specifically and exclusively from neurons has been shown to enhance LTP in rat and mouse hippocampal slices (Rosenberg et al., 2013). Also, a conditional deletion of SR gene in neurons lead to reduced dendritic arborization and spine density of pyramidal neurons of the somatosensory cortex, supporting a major role of neuronal D-serine over astroglial D-serine in this process (Balu and Coyle, 2012). Because D-serine dynamics are intermingled between neurons and astrocytes, any clear conclusion about the role of each source is difficult to draw, regardless of the available cell-specific genetic tools and metabolic poisoning. The cellular origin of D-serine, neuronal versus glial, could reflect upon adaptive mechanisms at individual or groups of synapses according to specific spatio-temporal dynamics and metabolic needs. This could be especially important in development given that D-serine metabolism is developmentally regulated (Puyal et al., 2006; Dun et al., 2008). It is already present during the first postnatal week (Kartvelishvily et al., 2006; Miya et al., 2008), yet, most glial cells in rodents, produced after birth, increase their number by sixfold to eightfold within the first three postnatal weeks (Bandeira et al., 2009; Ge et al., 2012) to establish appropriate astrocytic and neuronal network architectures. Although a significant number of astrocytes are largely generated during the first postnatal week, the fine processes of astrocytes that contact synapses in adulthood are not formed until several weeks later (Yang et al., 2013). The astroglial network, indicated by the interacting processes is also formed at later developmental stages (postnatal day 14–26). Thus, it is possible that neuronal and glial D-serine display differential onset during postnatal development. Accordingly, we can postulate that neuronal D-serine will first enter on stage and maintained during postnatal development and adulthood while glial D-serine would become available after the third postnatal week.

## RELEASE MECHANISMS OF D-SERINE

**FIGURE 2:**
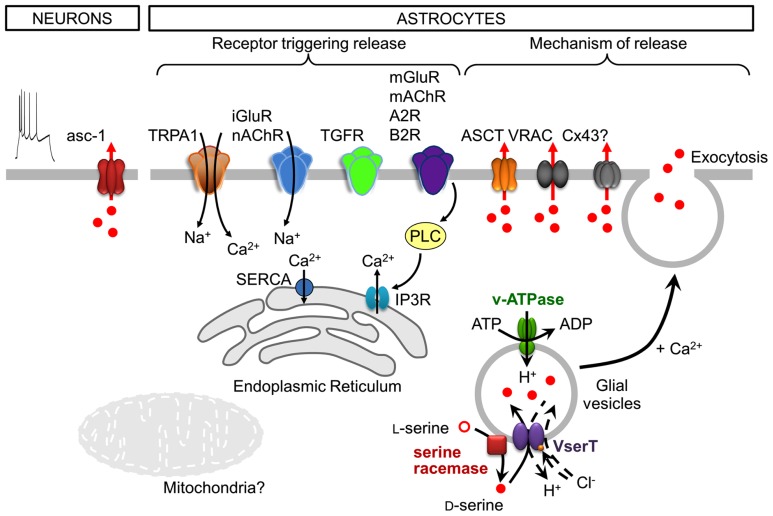
**Cell-type specific, neuronal and astroglial, stimuli, and mechanisms of D-serine release.** Following depolarization, neurons release D-serine from the cytosol through the transporter asc-1. Note that neuronal depolarization is shown by the action potential discharges (utmost left). Astrocytes express a plethora of functional receptors, the activation of which is coupled to the release of D-serine. In many cases this would include a downstream activation of phospholipase C (PLC). Astrocytes release D-serine through Ca^2^^+^- and SNARE-dependent exocytosis along with that ocurring through alternative non-exocytotic pathways including volume-regulated anion channels (VRAC), ASCT, and likely through connexin 43 hemichannels (Cx43). The release of glial D-serine by exocytosis requires its uptake into glial secretory vesicles. The vesicular D-serine transporter is proposed to be a D-serine/chloride co-transporter. A spatial association of SR activity and D-serine vesicular transport results in a local and functional efficient coupling between D-serine synthesis and uptake. See text for details.

As shown so far, D-serine can behave as a neurotransmitter and a gliotransmitter in the same area of the brain. The accurate NMDAR activation requires reliable and cell-specific release mechanisms depending on the physiological or pathological condition faced by the neuronal network. Different elements of the literature support the notion that neurons and astroglia use different molecular machinery and signaling pathways to release D-serine into the extracellular space (**Figures 1** and **2**).

The release of D-serine by astrocytes is mainly dependent on an increase in cytosolic Ca^2^^+^ and on SNARE (soluble N-ethylmaleimide-sensitive factor attachment protein receptor) proteins, indicating Ca^2^^+^-regulated exocytosis as a release mechanism. Indeed, disrupting Ca^2^^+^ signaling inside astrocytes reduces D-serine release not only from astrocytes in culture and in hippocampal slices (Mothet et al., 2005; Henneberger et al., 2010; Shigetomi et al., 2013), but also from astrocytes *in vivo* (Takata et al., 2011). Calcium certainly represents the most critical signaling aspect of D-serine release from astrocytes, even though the spatial and temporal dynamics of Ca^2^^+^ signals are still poorly defined. Several studies have provided evidence that the activation of glutamatergic and other G-protein coupled receptors (GPCR; see below) triggers the release of D-serine from astrocytes in culture and in brain slices. The activation of the GPCRs is associated with the recruitment of Ca^2^^+^ from the intracellular stores mainly via inositol-1,4,5-trisphosphate receptors (IP_3_R) located on the endoplasmic reticulum (ER; Zorec et al., 2012); the store is filled by the store-specific Ca^2^^+^-ATPase (SERCA). Electron and fluorescence microscopy analyses report that the ER is found beneath astrocytic plasma membrane, in close proximity to vesicles (Marchaland et al., 2008; Bergersen et al., 2012). Astrocytes appear to possess functional domains where Ca^2^^+^ increase occurs in spatial and temporal correlation with vesicular fusion events (Marchaland et al., 2008) allowing release of gliotransmitters. Although the ER and IP_3_Rs may provide the main route for Ca^2^^+^ signals, other intracellular organelles and receptors such as mitochondria and ryanodine receptors of the ER could also be involved in the build-up of cytosolic Ca^2^^+^ concentration that governs exocytotic D-serine release (Zorec et al., 2012). Indeed, mitochondria are involved in the Ca^2^^+^ signaling necessary for glial glutamate release (Reyes and Parpura, 2008) through the calcium uniporter, mitochondrial Na^+^/Ca^2^^+^ exchanger (Reyes and Parpura, 2008; Parnis et al., 2013) and mitochondrial permeability transition pore/cyclophilinD (Reyes and Parpura, 2008; Reyes et al., 2011). However, the involvement of mitochondrial Ca^2^^+^ signaling in the release of D-serine has not been shown. Ca^2^^+^ driving D-serine release could also come from the extracellular space through channel-mediated transmembrane Ca^2^^+^ fluxes in astrocytes. Astrocytic transient receptor potential A1 (TRPA1) channels contribute to basal Ca^2^^+^ signals which are required for D-serine release and can modulate LTP (Shigetomi et al., 2013; **Figure 2**).

Early studies have shown that D-serine release is triggered by agonists of the ionotropic and metabotropic glutamate receptors (iGluR and mGluR, respectively; Schell et al., 1995; Mothet et al., 2005; Martineau et al., 2008). More recent studies have now shown that many receptors for different neuroligands are coupled to the release of D-serine from astroglia (**Figure 2**). Accordingly, the activation of receptors for transforming growth factor (TGF)-β (TGFR; Diniz et al., 2012), bradykinin-type2 (B2R; Martineau et al., 2008), adenosine-type 2 (A2R; Scianni et al., 2013), ephrinB3 (Zhuang et al., 2010), and muscarinic or nicotinic acetylcholine receptors (mAChR and nAChR, respectively; Takata et al., 2011; López-Hidalgo et al., 2012; Lin et al., 2014) triggers the release of D-serine from astrocytes, as demonstrated using cultured astrocytes, and more intact preparations of brain slices or live animals. These studies clearly established that astrocytes express a plethora of functional receptors which activation is coupled to the release of D-serine; in many cases this includes a downstream activation of phospholipase C (PLC; **Figure 2**). Taken together, various molecular entities on the ER and the plasma membrane seem to provide for a complex astroglial Ca^2^^+^ excitability which can trigger D-serine release.

Fusion of vesicles with the plasma membrane is promoted by the formation of the SNARE complex which spans the vesicle and plasma membranes (Jahn and Fasshauer, 2012). The astroglial vesicle membrane contains synaptobrevin 2 (Sb2) and its homologue cellubrevin, either of which form the ternary SNARE complex with synaptosome-associated protein of 23 kDa (SNAP23) and syntaxin 1 present at the plasma membrane (reviewed in Montana et al., 2006). The vesicle fusion is triggered by an increase in cytosolic Ca^2^^+^, which presumably binds to vesicular synaptotagmins (Montana et al., 2006). Astroglial vesicles also possess proteins necessary for vesicular filling, such as the vacuolar type H^+^-ATPase (V-ATPase; Wilhelm et al., 2004; Martineau et al., 2013) which provides the proton gradient necessary for intravesicular loading of gliotransmitters via appropriate transporter(s). Cleavage of Sb2 and cellubrevin by tetanus neurotoxin causes a strong inhibition of Ca^2^^+^-dependent D-serine release (Mothet et al., 2005; Henneberger et al., 2010). Additionally, the blockade of D-serine vesicular uptake using a V-ATPase blocker inhibits D-serine release from astrocytes (Mothet et al., 2005). Indeed, based on electron microscopy (EM), D-serine accumulates in clear vesicles with a diameter of 36 nm in the perisynaptic processes of hippocampal and cortical astrocytes (Bergersen et al., 2012; Martineau et al., 2013). In the adult hippocampus, clear vesicles are organized in small groups of 2–15 vesicles preferentially located within 100 nm from the astrocytic plasma membrane (Bergersen et al., 2012), and observed at sites adjacent to neuronal elements bearing NMDARs (Bezzi et al., 2004). Thus, astrocytes possess small vesicles resembling those found at synaptic terminals (Harris and Sultan, 1995), albeit at lower density. It should be noted, however, that the vesicular size in astrocytes appears to be more diverse than that described in neurons, as Sb2-laden vesicles in live cultured astrocytes have been measured at ~300 nm (Malarkey and Parpura, 2011; Singh et al., 2014). In addition to Ca^2^^+^- and SNARE-dependent exocytotic release of D-serine from astrocytes, this gliotransmitter can be released via alternative non-exocytotic conduits at the plasma membrane, including volume-regulated anion channels (VRAC; Rosenberg et al., 2010), alanine–serine–cysteine transporter (ASCT; Ribeiro et al., 2002, but see Maucler et al., 2013) and likely through connexin 43 hemichannels (Stehberg et al., 2012; **Figure 2**).

Although the vesicular transporter for D-serine has not been identified, the transport of D-serine inside astroglial vesicles was recently characterized (Martineau et al., 2013). While glutamate transport is observed in both synaptic and astroglial vesicles, the transport of D-serine is specific to astroglial vesicles (Martineau et al., 2013). Its apparent affinity is ~7 mM, consistent with the affinity of vesicular inhibitory amino acid transporter for γ-aminobutyric acid, another neutral amino acid (Edwards, 2007; Martineau et al., 2013). Similar to glutamate, extravesicular chloride concentration modulates D-serine transport into astroglial vesicles, reaching the maximum activity at 4 mM. Because D-serine transport induces vesicular acidification and critically relies on chloride, the vesicular D-serine transporter is proposed to be a D-serine/chloride co-transporter (Martineau et al., 2013; **Figure 2**). A spatial association of SR activity and D-serine vesicular transport was observed, resulting in a functional coupling between D-serine synthesis and uptake (Martineau et al., 2013). Finally, D-serine and glutamate vesicular loading exert a mutual stimulation which indicates a functional crosstalk between the two transporters (Martineau et al., 2013). This synergy at vesicular level can only be explained if both transporters reside on the same vesicle, indicating the co-storage and thus the co-release of both gliotransmitters. However, the immunogold colabeling of D-serine and glutamate in the adult hippocampus did not reveal a population of vesicles containing both gliotransmitters (Bergersen et al., 2012); this seemingly disparate findings could be a result of the limited sensitivity of the immuno-EM. Nonetheless, the mechanism underlying the vesicular synergy between D-serine and glutamate uptake requires additional investigation. The possible co-storage of glutamate and D-serine, at least in a subpopulation of astroglial vesicles, points to the possibility of interdependence of their dynamics and modulatory functions at synaptic and extrasynaptic sites.

Release of D-serine as a neurotransmitter operates through a different mechanism than those described for a gliotransmitter. Consistent with D-serine absence in the lumen of synaptic vesicles (Martineau et al., 2013), D-serine is released by neuronal presynaptic elements from a cytosolic pool through the alanine–serine–cysteine transporter 1 (asc-1; Rosenberg et al., 2010, 2013; **Figures 1** and **2**). Asc-1 is a Na^+^-independent antiporter restricted to neurons which catalyzes neutral amino-acid hetero-exchange (Fukasawa et al., 2000; Nakauchi et al., 2000; Helboe et al., 2003; Matsuo et al., 2004), meaning that the release of D-serine is coupled to the uptake of another neutral amino acid. It is the main plasma membrane transporter mediating D-serine uptake (Rutter et al., 2007; and see the next heading of this review) in the post-synaptic neuronal elements (**Figure 1**).

Neuronal D-serine is mainly released in response to depolarization, induced by veratridine, a voltage-dependent Na^+^-channel activator, or by increased extracellular potassium concentration, both *in vitro* and *in vivo* (Rosenberg et al., 2010; **Figure 2**). Depolarization of cortical neurons induces a release of D-serine ten times more effectively than the glutamatergic AMPA (α-amino-3-hydroxy-5-methyl-isoxazole propionate) receptor activation (Rosenberg et al., 2010). Chelation of intracellular and extracellular Ca^2^^+^ does not affect depolarization-elicited D-serine release. In addition, inhibition of V-ATPase by bafilomycin A1 or cleavage of Sb2 by tetanus neurotoxin failed to inhibit D-serine release from neurons, excluding the vesicular release mechanism (Rosenberg et al., 2010). Finally, neuronal D-serine release elicited by addition of neutral amino acids, such as D-alanine or D-isoleucine, was independent of Na^+^ (Rosenberg et al., 2010, 2013), arguing for a hetero-exchange through asc-1, the only known Na^+^-independent D-serine transporter. Interestingly, D-isoleucine selectively interacts with asc-1 without affecting the Na^+^-dependent D-serine transporters ASCT1 and ASCT2 in HEK293 cells transfected to express various transporters (Rosenberg et al., 2013). The contribution of asc-1 was further confirmed by its inhibition with S-methyl-L-cysteine, which impaired D-serine release evoked by L-serine; however, L-asparagine, a substrate of ASCT2, did not induce D-serine release *in vivo* (Maucler et al., 2013).

## CONTROL OF EXTRACELLULAR D-SERINE CONCENTRATION

Concentration of extracellular D-serine is affected by two types of antiporters: the Na^+^-dependent ASCT1 and ASCT2 (Ribeiro et al., 2002; Maucler et al., 2013), and the already discussed Na^+^-independent asc-1 (Rutter et al., 2007; Rosenberg et al., 2013). These antiporters mediate the hetero-exchange of small neutral amino acids and finely regulate D-serine concentration in the extracellular space (**Figure 1**).

ASCT1 and ASCT2 (also known as SATT and AAAT; products of *SLC1A4* and *SLC1A5* genes, respectively) are members of the alanine–serine–cysteine (ASC) family of Na^+^-dependent substrate exchangers (see Kanai et al., 2013 for review). These two ASC family members show highest affinity for L-alanine, L-serine, L-cysteine, and L-threonine, and stereoselectivity for L- over D-amino acids (Arriza et al., 1993; Utsunomiya-Tate et al., 1996). Additionally, ASCT2 transports L-glutamine and L-asparagine with high affinity (Utsunomiya-Tate et al., 1996). ASCT1/2 are both expressed in astrocytes and neurons (Bröer et al., 1999; Weiss et al., 2001; Yamamoto et al., 2003, 2004; Gliddon et al., 2009; Shao et al., 2009). ASCT1 appears to be the predominant system for uptake of L-serine in cultured neurons (Yamamoto et al., 2004). Despite their low affinity for D-serine (Arriza et al., 1993; Utsunomiya-Tate et al., 1996), a transporter with specificities resembling that of ASCTs was shown to be responsible for D-serine uptake in cultured neurons and astrocytes (Ribeiro et al., 2002; Shao et al., 2009), and also *in vivo* (Maucler et al., 2013). Because an early study found that ASCT1 practically does not recognize D-serine (Shafqat et al., 1993), the uptake of this amino acid was assumed to be mediated by ASCT2. However, the recent overexpression of ASCT1 and ASCT2 in HEK293 cells showed similar D-serine uptake for both transporters (Rosenberg et al., 2013).

Asc-1 (encoded by *SLC7A10* gene) is a member of the SCL7 solute carrier family (see Fotiadis et al., 2013 for review). As indicated earlier, asc-1 is a Na^+^-independent antiporter allowing permeation of small neutral amino acids such as glycine, L-alanine, L-serine, L-threonine, and L-cysteine with high affinity (Fukasawa et al., 2000; Nakauchi et al., 2000). Contrary to ASCTs, asc-1 displays a low stereospecificity and also transports small neutral D-amino acids, in particular D-serine with a high affinity (*K*_m_ ~50 μM; Fukasawa et al., 2000). Asc-1 operates preferentially in an exchange mode, but can also work as a facilitative diffuser (Fukasawa et al., 2000; Rosenberg et al., 2013). Asc-1 is widely expressed in the brain, being restricted to neuronal structures (Helboe et al., 2003; Matsuo et al., 2004; Shao et al., 2009). Genetic ablation of asc-1 in mice results in a 70–80% reduction in the D-serine uptake in the forebrain and cerebellar synaptosomes, indicating that asc-1 is the main plasma membrane D-serine transporter (Rutter et al., 2007). These asc-1 knockout mice exhibited tremor, seizures, and early postnatal death consistent with the over-activation of NMDARs due to elevated extracellular D-serine levels (Xie et al., 2005). Because more specific glycine transporters (GlyTs) exist (Eulenburg et al., 2005), asc-1 is expected to affect to a lesser extent extracellular glycine levels. Asc-1 plays a critical role in the extracellular disposition of D-serine and in the regulation of neuronal excitability. Yet, it is not clear how asc-1 and ASCT1/2 transporters are working together to constraint extracellular D-serine diffusion and how they would represent a diffusion barrier that determines the occupancy of NMDARs by D-serine.

## SPATIAL AND TEMPORAL CONSTRAINTS OF D-SERINE FUNCTION

Neuronal D-serine as well as glycine can be released by asc-1 upon application of neutral amino acids such as D-isoleucine (Rosenberg et al., 2010, 2013). Interestingly, D-isoleucine mimics the activation of asc-1 by physiological extracellular substrates (L-alanine, L-serine, and L-cysteine) present in the brain (Rosenberg et al., 2013). Thus, the neuronal release pathway could be responsible for setting a basal D-serine concentration in the extracellular space over a slow time scale and large, if not global, brain volume, and, thus, allowing for basal NMDAR activity. In stark contrast, exocytosis of D-serine from astrocytes could provide for higher focal D-serine concentration on a shorter time scale, allowing for a precise spatial and temporal boost in NMDAR activation (**Figure 1**). However, it is still unknown whether D-serine is released by astrocytes as well as neurons at specialized zones, and where these zones might be located. Some inferences to the location of this process can be drawn from electrophysiology results, however. Recently, D-serine and glycine have been shown to activate distinct populations of NMDARs in the hippocampus, with D-serine binding to synaptic GluN1/GluN2A-containing NMDARs and glycine to the extrasynaptic GluN1/GluN2B-containing counterparts (Papouin et al., 2012). Different NMDAR populations may trigger different forms of plasticity (Liu et al., 2004). Accordingly, D-serine depletion blocked LTP induction while long-term depression (LTD) required both glycine and D-serine (Papouin et al., 2012). The regional occupancy of NMDARs by the coagonists matches the preferential affinity of synaptic NMDARs for D-serine and extrasynaptic NMDARs for glycine. However, it is not known which release/uptake mechanisms provided this functional compartmentalization and how glutamate may activate extrasynaptic sites together with glycine. Because the level of D-serine vary across brain regions (Schell et al., 1997), the relative contribution of the different coagonists is likely to be heterogeneous throughout the brain. Accordingly, different contemporary studies challenge the appealing model of exclusive roles of D-serine and glycine at synaptic and extrasynaptic NMDARs, respectively. Indeed, both neuronal D-serine and glycine released through asc-1 were overlapping to regulate the NMDARs at CA3-CA1 glutamatergic synapses (Rosenberg et al., 2013). Furthermore, the identity of the coagonist of NMDAR at synapses in the lateral nucleus of the amygdala was determined by the level of synaptic activation (Li et al., 2013). Tonic activation of NMDARs in the amygdala under low activity conditions was supported by ambient D-serine, whereas glycine may be released from astrocytes in response to afferent stimulation. Strikingly, GluN1/GluN2A/GluN2B tri-heteromers have recently emerged as the preferentially NMDARs localized at synapses in the adult forebrain (Soares and Lee, 2013; Hansen et al., 2014). Thus, the nature of the coagonist may not correspond to spatial segregation of the NMDAR subunits, but could rather be determined by synaptic inputs/afferents. Understanding the orchestration between the different cellular sources for D-serine and unraveling the location/existence of specialized release/uptake zones for astroglial and neuronal D-serine and glycine will ultimately help to elucidate the more precise roles of these amino acids as critical players in the NMDAR-mediated synaptic transmission and plasticity.

## CONCLUDING REMARKS AND FUTURE DIRECTIONS

Important advances have been made since the discovery of the presence of D-serine in the brain in the early 1990s. There has been considerable progress in our understanding of the metabolism, and signaling pathways involving this atypical and novel brain messenger with implications for the pathophysiology of human diseases. Even though there have been developments of small molecules modulating D-serine actions that could be used soon in therapy for the treatment of cognitive deficits associated with aging, mood disorders and schizophrenia (Ferraris and Tsukamoto, 2011; Panizzutti et al., 2014), there are still many basic cell biology questions that remain to be answered, e.g., how D-serine and glycine operate together at glutamatergic synapses. It appears as that in the brief period of about two decades we went over the initial glycine era and then the D-serine era to finally enter the composite era in which glycine and D-serine can act as coagonists in NMDAR-mediated synaptic transmission. Such an inclusive approach holds promise to aid unveiling the complexity of glutamatergic neuro- and glio-transmission.

## Conflict of Interest Statement

The authors declare that the research was conducted in the absence of any commercial or financial relationships that could be construed as a potential conflict of interest.
